# Supplementary material for: Optimising access to vocational rehabilitation through multiple sclerosis charities: Protocol for a feasibility randomised controlled trial
Source: PLoS One. 2025 Jun 27;20(6):e0325570. doi: 10.1371/journal.pone.0325570 (PMC12204529; doi:10.1371/journal.pone.0325570)
Supplement: S2 File — (DOCX) [file pone.0325570.s002.docx]

Optimising access to vocational rehabilitation through multiple sclerosis charities: A feasibility randomised controlled trial

**Research Team**

Dr Blanca De Dios Pérez, University of Nottingham

Professor Denise Kendrick, University of Nottingham

Professor Roshan das Nair, University of Nottingham

Professor Nikos Evangelou, University of Nottingham

Mr Ian Newsome, Lead PPI

Professor Kathryn Radford, University of Nottingham

**Introduction**

Multiple Sclerosis (MS) is the most common chronic neurological condition affecting young adults [1]. Women are two times more likely to develop MS than men, and currently, MS affects over 150,000 people in the UK [2]. People are usually diagnosed between 20 and 40 years of age, the prime working years of an adult [3].

MS is characterised by progressive damage to the Central Nervous System (CNS), producing plaques in the brain and spinal cord [4]. These lesions are caused by the loss of myelin sheaths which leads to inflammation and axonal damage (or loss) [1].

Due to the damage to the brain, people with MS can present with a range of physical and cognitive problems. The most common physical problems presented in MS are gait difficulties that can lead to people needing a wheelchair and balance disorders, which lead to an increased risk of falls [5]. In fact, after 15 years with the condition, approximately 50% of people with MS need support walking [6].

Furthermore, up to 65% of people with MS present some level of cognitive impairment [7]. Although significant variability exists in the types and intensity of impairments MS can cause, memory and attentional problems are the most frequently reported in this population [7–9].

To this array of physical and cognitive impairments, MS is also characterised by the presence of “invisible symptoms”, which are symptoms not visible to others. Some invisible symptoms are fatigue, pain, bladder and bowel problems, and vision difficulties, among others [10]. From these, fatigue is the most reported symptom in MS (approximately 90% of people with MS) [11].

The range of physical, cognitive, and psychological problems that people with MS can present increases the personal and professional challenges that people with MS experience at work [12].

Vocational rehabilitation (VR) aims to help people with illness or disabilities to remain, return to or find new employment. We have previously developed a VR intervention to support people with MS to remain in paid employment called MSVR (multiple sclerosis vocational rehabilitation) [13]. We have tested this programme in a community setting [14], and within the NHS [15]. However, we found barriers (e.g., lack of staff, service structure, etc.) to deliver this programme within a healthcare setting [15,16]. Patient and public involvement (PPI) representatives suggested testing this programme within the services provided by MS charities.

Before conducting a larger study, the feasibility of delivering VR through MS charities needs to be established. This study was informed by the Medical Research Council (MRC), which provides guidance for developing complex interventions [17].

**Aims**

The primary aim is to assess the feasibility and acceptability of conducting a multicentre, parallel-group RCT comparing MSVR delivered to participants recruited from MS charities with existing services offered by the charities alone. More specifically, we will focus on:

1. Recruitment: To assess the length of time to recruit the sample planned, the recruitment rate, the proportion of eligible patients recruited, reasons for non-recruitment, and the appropriateness of study characteristics such as inclusion and exclusion criteria.
2. Retention and engagement: To estimate the proportion of participants lost to follow-up and the reasons for loss to follow-up, withdrawal rates, data completeness, and participants’ compliance with MSVR and identify factors affecting compliance.
3. Intervention delivery: To assess feasibility of delivering up to 10 hours of VR over 6 months, intervention fidelity, intervention adherence, feasibility of delivering VR alongside existing services of the charities, and practical issues related to intervention delivery.

The secondary aims are to determine parameters for a sample size calculation for a future large-scale RCT and explore the mentoring and training needs of the MSVR champions (i.e., employees from MS charities recruited to deliver the intervention) recruited for the study.

This study includes an embedded mixed methods process evaluation, the aims of which are to understand acceptability and usefulness of:

- MSVR for people with MS, employers, and MSVR champions.
- study procedures to MS charities and participants.
- MSVR training manual, sessions, and mentoring support.

**Methods and Analysis**

This is a multicentre, feasibility, parallel-group RCT with an embedded mixed methods process evaluation. Participants will be randomised (1:1 ratio) to receive either MSVR plus usual care (UC) or UC alone. This study will be reported following the CONSORT 2010 statement: extension to randomised pilot and feasibility trials [18].

**Eligibility criteria**

The study will recruit people with MS (1) between 18 and 65 years of age, (2) who can give informed consent, (3) who can communicate in English, and (4) who are in paid employment (including self-employed). This study will exclude participants who are planning to retire within the next 12 months or are currently receiving vocational rehabilitation support.

If a participant with MS consents to involving their employer in the intervention, they will be included if they are (1) between 18 and 65 years of age, (2) can give informed consent, (3) can communicate in English, and (4) is currently employing a person with MS. We will consider the line manager or a human resources representative as the employer of the person with MS. There are no exclusion criteria for the employers.

MSVR champions (i.e., employees from MS charities recruited to deliver the intervention) will be asked to complete a consent form to complete a demographic and skills questionnaire and participate in an interview at the end of the study.

**Setting and locations**

The University of Nottingham will coordinate this study, which will involve up to six MS charities. The charities will be purposively recruited based on their attributes (e.g., organisation size, resources, and services available), and interest in optimising their existing services. The intervention will be delivered remotely via Microsoft Teams and/ or telephone, according to the participant’s preferences. The study will be conducted between June 2025 and November 2027.

**Recruitment**

Participants with MS will be recruited through the participating MS charities over a 10-month period. The organisations will share information about the study using a multi-pronged approach to reach a broader range of people with MS. Therefore, the study will be advertised on their helplines, social media, websites, and bulletins.

The initial approach to potential participants with MS will be made by the MS charities employees through their helpline support, newsletters, and events. They will provide information about the study using a study advert and participant information sheet (PIS). People with MS declining the opportunity to participate will be asked for a reason for not participating, but they do not have to reply.

We will develop a recruitment matrix to ensure we recruit a diverse sample of participants to the feasibility trial. The matrix will include information such as ethnicity, socioeconomic status (based on deprivation index), gender, age, and employment characteristics.

People with MS will also have the opportunity to read about the study online (e.g., social media, websites, bulletins). If a person with MS is interested in participating, they will be asked to complete an expression of interest with the charity worker to consent for their details to be shared with the researcher or to contact the lead researcher via telephone or email.

Those people with MS who are interested in participating in the study will be told that they can include their employer (e.g., line manager or human resources representative) in the intervention to receive information and advice about MS at work. It is not mandatory for the person with MS to include their employer, and if the person with MS does not consent to include their employer, the employer will not be contacted.

**Screening and informed consent**

Potential participants with MS will be contacted via telephone to be screened against the inclusion criteria by the lead researcher (BDP). A screening log will be used to monitor and record information about participants screened and reasons for not being recruited.

**Intervention: MSVR**

The full description of the intervention is presented elsewhere [13]. Due to feedback from previous studies [15], the intervention was extended to support the person with MS over six months (instead of the original three months).

MSVR will be delivered by employees from MS charities currently working within MS charities supporting people with MS with advocacy or information provision. These employees have a wide range of professional backgrounds, expertise in MS (including symptoms and progression), skills with active listening, and have been trained to handle difficult conversations. The workers from MS charities, called “MSVR Champions”, will receive training and mentoring in preparation for the trial.

MSVR for people with MS involves an initial interview (including vocational goal setting), followed by up to 10 hours of individually tailored support according to need. The support will focus on supporting the person with MS to meet their vocational goals by addressing topics such as:

- Understanding MS
- Advice on reasonable adjustments (e.g., modification to the work environment or duties to accommodate the impact of MS)
- Support requesting reasonable adjustments
- Fatigue management
- Managing cognition at work
- Information about legal rights
- Disclosure (i.e.., telling the employer or colleagues about the MS diagnosis)
- Long-term career planning (i.e., exploring alternative career options)
- Managing mood difficulties
- Signposting to local and national resources.

The intervention addresses the individual needs of the person with MS and offers advice remotely (e.g., via telephone, videoconference, or email). The sessions will be arranged at a time convenient for the person, and the support needed will vary according to the complexity of their workplace difficulties. Participants will receive a brief written summary of the content discussed in each session via email with further resources to complement the knowledge gained.

MSVR for the employers will involve an initial interview (approximately 30 minutes) to understand their experiences supporting the employee with MS and up to 4 hours of support over the six-month intervention. The employer’s intervention includes:

• Signposting to relevant organisations.

• Educational resources.

• Information about MS and invisible symptoms.

• Support with providing reasonable adjustments.

• Legal responsibilities (Equality Act).

After each session, the employer will receive an email summarising topics discussed and, when required, a list of actions they should complete before the next session.

**Control Condition**

Participants (MS and employers) in the control group will receive resources from the MS charities' websites, helplines, and events. Due to the nature of the study, usual care will vary between participants in the control group. However, no MS charity recruited for this study will have a specialist vocational rehabilitation service (and none should have indicated that they will be creating such a post soon).

Participants (MS and employers) in the control group will interact with members from the MS charity who have not been trained and are not involved in MSVR delivery to reduce the potential for contamination.

At the end of the study, participants with MS in the control group will receive the “Work and MS: An Employee’s Guide” booklet from the MS Society, which contains information about MS and work. PPI representatives suggested this to reduce the burden of being allocated to the control group and encourage the completion of follow-up questionnaires.

*Concomitant Therapy*

Participants will continue to use services from social care, the National Health Service (NHS), and other third-sector organisations alongside MSVR. Information on any concomitant therapy received will be collected using a resource use questionnaire.

**Monitoring and Mentoring**

An Occupational Therapist (OT) (JH) with extensive working experience in VR for people with long-term health conditions will offer monthly mentoring sessions to the MSVR champions.

The mentoring sessions will be conducted in groups via videoconference to discuss cases, confidentiality issues, contamination, and challenges during the intervention delivery. MSVR champions will also be able to request further mentoring if a query appears outside the mentoring sessions.

The OT will complete a mentoring record form to capture topics addressed and questions raised by the MSVR champions during the mentoring sessions to gain further understanding of the skills needed to deliver MSVR and additional training needs.

**Primary and secondary outcomes**

The primary outcomes assess the feasibility and acceptability of delivering MSVR within the context of MS charities by measuring recruitment, retention and engagement, and intervention delivery.

Secondary outcomes refer to determining the parameters for a fully powered trial, including selecting a primary outcome measure, sample size estimation, intervention costs, and identifying intervention improvements and further training needs of MSVR champions. The feasibility outcomes are summarised in Table 1.

**Table 1 Feasibility outcomes and measures**

| Feasibility objectives | Outcomes and measurement |
| --- | --- |
| To assess the feasibility and acceptability of MSVR | **Recruitment**  Length of time to recruit the sample will be calculated using the date of the first and last participant recruited to the feasibility trial  Number of participants with MS screened, meeting the inclusion criteria, recruited and randomised.  Number and reasons of participants not fully meeting the inclusion criteria.  **Retention and engagement**  Number of participants lost to follow-up  Number of participants who withdraw from the intervention  Amount of missing data from questionnaires  Number of participants who complete the initial interview and at least two intervention sessions.  **Intervention delivery**  Hours of support delivered, and number of sessions attended by the participants with MS and employers.  Number of participants who do not attend sessions and number of early dropouts  Hours of support delivered per intervention component and number of participants completing the intervention as structured.  Number of mentoring sessions attended by each MSVR champion.  MSVR champions attendance to training and barriers to delivering the intervention within existing services.  **Acceptability**  Feedback on the intervention content, delivery mode, and structure. |
| To plan for a fully powered trial] | **Selection of primary outcome measure**  Determined by importance of different outcome measures, response rates, and missing data of outcome measures on questionnaires, and preliminary effect size estimates of the outcome measures.  **Sample size**  To estimate the standard deviation of the continuous primary outcome measure with a 95% confidence interval, including lost to follow-up and data completeness.  **Intervention cost**  Cost of MSVR champions and mentor’s time. |

**Outcome measures**

Outcomes measures will be assessed at the end of the intervention and 3-, 6-, and 12-months post-intervention. The measures collected and time points have been summarised in Table 2.

**Table 2 Summary of assessments and time points**

| Activities / Measures | Assessment and time-point | | | | | | |
| --- | --- | --- | --- | --- | --- | --- | --- |
|  | **Screening** | **Baseline**  **(A1)** | **Intervention** | **End of Intervention**  **(A2)** | **3 months FU** | ***6 months***  ***FU*** | ***12 months***  ***FU*** |
| Screening for eligibility | x |  |  |  |  |  |  |
| Completion of consent form | x |  |  |  |  |  |  |
| Demographic information | X |  |  |  |  |  |  |
| Randomisation | x |  |  |  |  |  |  |
| Goal Attainment Scaling |  | x | **MSVR Intervention** | x | x | x | x |
| 36-item short form health survey |  | x |  | x | x | x | x |
| Perceived Difficulties Questionnaire |  | x |  | x | x | x | x |
| Work Productivity and Activity Impairment Questionnaire for MS |  | x |  | x | x | x | x |
| Modified Fatigue Impact Scale -5 Items version |  | x |  | x | x | x | x |
| EQ-5D-5L |  | x |  | x | x | x | x |
| Generalised anxiety disorder scale |  | x |  | x | x | x | x |
| Patient health questionnaire 8-item scale |  | x |  | x | x | x | x |
| General self-efficacy scale |  | x |  | x | x | x | x |
| Workplace Accommodations questions |  | x |  | x | x | x | x |
| Resource use questionnaire |  | x |  | x | x | x | x |
| Adverse events |  |  | x |  |  |  |  |
| Intervention cost (training, mentoring, intervention delivery) |  |  | x |  |  |  |  |
| Acceptability of MSVR (interviews with people with MS and employers) |  |  |  | x |  |  |  |
| Acceptability of training (MSVR champion questionnaire) | x |  |  |  |  |  |  |
| Acceptability of MSVR and mentoring (interviews with MSVR Champions) |  |  |  | x |  |  |  |
| A=Assessment; FU= Follow-up; EQ-5D= European Quality of Life- 5D; MS= multiple sclerosis; MSVR= Multiple Sclerosis Vocational Rehabilitation | | | | | | | |

The outcome measures will be collected using REDCap, or by post or telephone if a participant cannot access an electronic device. The acceptability measures (i.e., interviews) will be collected remotely via telephone or Microsoft Teams. To minimise missing data, we will set up a REDCap email notification to automatically remind participants to complete the questionnaires 2 weeks from the due date.

Participants completing the questionnaire by post or telephone will be contacted by a member of the research team via email or telephone to remind them to complete the questionnaire. If needed, a second copy of the booklet of questionnaires will be sent by post. Participants will receive priming calls from a member of the research team to remind them to complete the questionnaires.

*Participants with MS*

This feasibility study has two candidate coprimary outcome measures for a future definitive trial: (1) health-related quality of life as measured by the 36-item short form health survey (SF-36) [19] and (2) work productivity, measured using the Work Productivity and Activity Impairment (WAIP) for people with MS [20], extending the recall period from seven days to four weeks. These measures have been identified as relevant in other studies about MS and employment [21,22].

The SF-36 measures eight health constructs relating to physical and mental health. This measure has a high internal consistency (Cronbach’s alpha >0.80) and good test-retest reliability (>0.70). The WAIP:MS measures absenteeism and presenteeism, productivity loss and activity impairment. Higher scores in the WAIP:MS represent a higher impact of MS at work and conducting everyday activities.

The European Quality of Life with five dimensions (ED-5D-5L) [23] will assess psychological and health-related quality of life, focusing on mobility, self-care, usual activities, pain/discomfort, and anxiety/ depression. Cognitive difficulties will be measured with the perceived deficits questionnaire (PDQ) [24,25], a 5-point Likert scale 20-item questionnaire with four sub-scales with a 0.93 Cronbach alpha. The sub-scales have good reliability and internal consistency: Cronbach’s alpha .78

(attention/concentration), .84 (planning/organization), .83 (retrospective memory),

and .76 (prospective memory) [26]. Fatigue will be assessed using the modified fatigue impact scale 5 (MIFS-5), a 4-point Likert scale with five items [27]. The General Self-Efficacy Scale (GSES) assesses the participants’ belief in their ability to complete tasks successfully [28]. Two brief questionnaires will assess mood: the Generalised Anxiety Disorder Scale (GAD-7) [29] and the Patient Health Questionnaire (PHQ-8) [30].

Vocational goals will be measured using the goal attainment scale (GAS) [31] to ascertain the impact of the intervention on the vocational goals set by participants at the beginning of the study. Workplace accommodations will be assessed using a series of binary questions (yes/no) about support received at work.

Finally, a resource use questionnaire will be developed to record the healthcare utilisation and additional services and support accessed during the feasibility trial.

*Employers*

Employers will be asked to complete three questions regarding their knowledge of MS, confidence in managing the employee with MS at work, and confidence in their ability to solve future problems at work.

*MSVR Champions*

MSVR champions will be asked to complete the educational course assessment toolkit (EDUCATOOL) [32]. A 12-item questionnaire to evaluate the training and learning experience of the MSVR champions during the study.

**Progression Criteria to the Future Trial**

Progression criteria will be assessed based on recruitment and follow-up rates after 4 months of recruitment based on a traffic light system of green (go), amber (review), and red (stop) [33]. The start of recruitment is defined as the first participant recruited into the trial.

Recruitment criteria will be assessed over months 3-4 to allow recruitment rates to stabilise. The follow-up criteria will be assessed at the end of the intervention. Feasibility will be demonstrated if:

- At least six people with MS are recruited per month across all sites (green), at least three but less than six (amber), or less than three (red).
- Percentage of participants randomised to the MSVR arm who complete the intervention (initial interview and at least one session), at least 80% green, at least 40% but less than 80% (amber) or less than 40% (red).
- The follow-up rate criterion is assessed by the percentage of people who complete the end of intervention survey with at least 80% (green), at least 65% but less than 80% (amber) or less than 65% (red).

If the rescue plan is not effective (e.g., progression criteria measures do not progress to amber or green), and any criteria are graded as red, we will not progress to the definitive trial. The findings from the progression criteria stages throughout the trial will be used to inform the full-scale future trial.

**Sample size**

A pragmatic target of 60 participants with MS (30 in each arm) was estimated based on personal communications with the MS charities recruited for the study and experiences from previous studies [14,15]. This figure aligns with recommended sample sizes for feasibility trials to estimate a parameter for a future RCT [34–36]. This sample size will provide sufficient data to achieve the study aims mentioned above.

Participants with MS will be asked if they are interested in recruiting their employer to the intervention, where they will receive information and advice about MS and employment. Based on previous research studies [14,15], we anticipate that approximately 20% of participants will consent to include their employer.

**Randomisation**

Following the screening procedures, eligible participants who want to participate will receive an email with an electronic consent form using REDCap. Following the completion of the consent form, participants will have the opportunity to complete the baseline assessment electronically (using REDCap) or over the telephone with the lead researcher (BDP).

After completing the consent form and baseline assessment, MS participants will be automatically randomised (1:1 ratio) to MSVR plus usual care (UC) or UC, using adaptative randomisation with minimisation through a computer. The MSVR champions will receive an automatic email informing them of the group allocation.

**Blinding**

Due to the nature of the intervention, the MSVR champions and the mentor will not be blinded to the intervention group of the participants. Participants will not be blinded to the intervention group allocation. The teams at the MS charities will not be able to predict allocation group when referring participants to the study.

Follow-up data will be collected, wherever possible, using REDCap. For participants who prefer to complete the questionnaire via post or telephone, a researcher, blinded to allocation group will collect the data. A form will be developed to record whether the researcher was unblinded during the data collection process.

The researcher supporting the recruitment of participants and conducting the data analysis will be blinded to the group allocation. Other research team members not involved in the direct management of the feasibility trial will remain blinded to group allocation until data collection concludes.

**Intervention adherence and fidelity measures**

Intervention adherence will be measured using the “intervention content forms”. MSVR champions will be trained to complete an intervention content form at the end of each session, reporting the modality of the session, topics discussed, length of time spent discussing each topic, and time spent by MSVR champion completing actions after the session (e.g., liaison with other professionals, reviewing forms, etc). Additionally, an “intervention summary form” form will capture the number of sessions completed by the participants and the number of sessions booked and cancelled (and reasons, if provided).

An intervention fidelity checklist will monitor whether the MSVR champions deliver the intervention as intended. MSVR champions will be asked to video or audio-record 10% of the intervention sessions to explore whether the intervention is being delivered as intended. The lead researcher will analyse the session's content compared to the data recorded in the “session content form” to identify discrepancies.

**Reporting and management of adverse events**

Serious Adverse Events (SAE) involve any untoward occurrence that results in death, is life-threatening, requires hospitalisation or prolongation of existing hospitalisation, results in persistent or significant disability or incapacity, or is otherwise considered medically significant by the investigator. Due to the nature of the intervention, we do not anticipate SAE. MSVR champions will be encouraged to discuss with participants whether they have experienced any problems or accidents at work based on the information provided during the intervention.

Related unexpected serious adverse events (RUSAE) will be any SAE where, in the opinion of the Principal Investigator (PI), the event was considered to be (1) “related” that is, it resulted from the administration of any of the research procedures, and (2) “unexpected” that is, the type of event is not listed in the protocol as an expected occurrence. In the case of this study, it could involve accidental injury resulting from workplace adaptations recommended by the MSVR champion, or workplace accidents resulting in injury requiring hospital treatment.

This data will be collected by self-report (participant questionnaires and ad-hoc CRFs, or via sites notifying a member of the research team). Any event that, in the opinion of the PI, is “related” (resulted from the administration of the research procedure) and “unexpected” (the type of event is not listed as an expected occurrence) will be reported to the research ethics committee within 7 days of being informed of such events. Fatal and life-threatening events will be reported no later than 3 calendar days after the sponsor or PI is first aware of the event. Any other additional relevant information will be reported within 7 calendar days of the initial report.

**Process Evaluation**

To refine the study procedures and intervention for a future trial, we will seek the views of participants with MS (in the intervention and control group), employers, and MSVR champions. Participants will be recruited using purposive sampling to recruit participants with diverse employment characteristics, participants who dropped out, and participants who did not request support. A research team member will conduct the interviews via telephone or video conference (e.g., Microsoft Teams).

At the end of the intervention, semi-structured interviews with the participants with MS (n=10), employers (n=5), and MSVR champions (n=5) will be conducted to explore the acceptability of MSVR, factors affecting their engagement in the intervention, acceptability of support received, their views on what attributes of the intervention where most beneficial for them, and views on how to improve the intervention for a future larger trial. Participants with MS will be asked what can be done in a future trial to encourage employer recruitment during the semi-structured interviews.

The interviews will also explore the impact of the mentor's support for delivering the intervention, to understand whether this support is needed for the long-term sustainability of the intervention within the MS charities' setting. They will also examine whether participants allocated to the control group actively sought support with employment elsewhere and whether they took self-directed action towards their vocational goals.

**Study management**

BDP has the overall responsibility for the study and will receive support from the co-investigators (KR, DK, RdN, NE) to manage and coordinate the study. The steering committee, including academics and PPI representatives, will meet every two months to discuss progress.

**Data analysis**

The study's quantitative data will be analysed using SPSS version 27.0 (Statistical Package for Social Sciences) following a predefined statistical analysis plan.

*Recruitment*

Data related to the number of eligible people, recruited, retained for the study (i.e., completion of interview and at least one intervention session), and completion rates of questionnaires will be analysed using descriptive statistics with 95% confidence intervals (CI) (where applicable).

*Demographic and baseline characteristics*

Demographic and baseline characteristics will be presented using descriptive statistics such as mean (standard deviation), median (interquartile range), or percentages to represent frequency. The type of descriptive statistic will vary according to the type of variable (e.g., continuous, ordinal, dichotomous, etc.). We will present both the overall and within groups descriptive statistics. We will explore the level of MS severity among participants recruited in the intervention and control group to understand which participant groups are expressing an interest in this support.

*Feasibility measures*

We will use the CONSORT diagram to report data regarding eligible individuals, participants screened, recruited (per month and per site), randomised, and lost to follow-up.

We will report the time to recruit the target sample, possible recruitment issues, the number of participants who book and attend the initial interview, the number of sessions booked and cancelled, hours of support received, topics addressed, withdrawal and follow-up rates, reasons for dropout (if provided), and the number of employers recruited. The hours of support received per participant will be used to determine the workforce needed for a future RCT.

Participants who drop out during the study will be asked (where possible) why they are dropping out and if they are still interested in completing the end of intervention interview and remaining assessments for the trial.

*Outcome measures*

All outcome measures will be summarised using descriptive statistics (as reported for the demographic characteristics) for all time points for the intervention group and control.

*Economic evaluation*

The cost of delivering MSVR will be estimated from the time MSVR champions spent participating in the training, attending mentoring sessions, and providing the intervention (measured with the session content forms). Data regarding the hourly rate of MSVR champions will be obtained from the MS charities participating in the feasibility trial.

We will also estimate the cost of the mentoring support based on the hourly rate of the mentors and hours of training, mentoring, and administrative support provided.

*Interviews*

The interviews will be digitally audio-recorded, transcribed verbatim, and analysed on NVivo v14.0. Interviews will be analysed thematically [37] using a hybrid deductive and inductive approach to develop codes and themes [38]. This analysis involves five steps (familiarisation with the data, generation of initial codes, generating themes, reviewing potential themes, and defining and naming themes) [39]. Any disagreements will be discussed with a third researcher (RdN).

**Patient and public involvement**

A lead PPI representative (White British Man, diagnosed with MS for 30 years, currently not working) has been co-developing this research programme since its inception and will be involved in the study governance and interpretation of the findings.

The development of MSVR included evidence from available literature and semi-structured interviews with people with MS, employers, and healthcare professionals [13]. Additionally, the idea from this research project resulted from discussions with people with MS and workers from MS charities that suggested the context of MS charities as a novel idea to offer employment support to people with MS and their employers.

**Dissemination**

The findings from this feasibility trial will be presented at national and international conferences, published in peer-reviewed journals, and disseminated in webinars and newsletters of the participating charities.

**Funding**

This study was funded by the UK MS Society (Ref:184)

**References**

[1] Goldenberg MM. Multiple sclerosis review. P T 2012;37:175–84.

[2] MS Society. MS Prevalence Report. 2020.

[3] Rumrill PD, Koch LC, Wohlford S. Job retention strategies for individuals with multiple sclerosis. J Vocat Rehabil 2013;39:127–35. https://doi.org/10.3233/JVR-130650.

[4] Trapp BD, Ransohoff RM, Fisher E, Rudick RA. Neurodegeneration in multiple sclerosis: Relationship to neurological disability. Neuroscientist 1999;5:48–57. https://doi.org/10.1177/107385849900500107.

[5] Gandolfi M, Munari D, Geroin C, Gajofatto A, Benedetti MD, Midiri A, et al. Sensory integration balance training in patients with multiple sclerosis: A randomized, controlled trial. Multiple Sclerosis Journal 2015;21:1453–62. https://doi.org/10.1177/1352458514562438.

[6] Navikas V, Link H. Review: Cytokines and the pathogenesis of multiple sclerosis. J Neurosci Res 1996;45:322–33.

[7] Rao. Neuropsychology of Multiple Sclerosis. Curr Opin Neurol 1995;8:216–20.

[8] Chiaravalloti ND, DeLuca J. Cognitive impairment in multiple sclerosis. Lancet Neurol 2008;7:1139–51. https://doi.org/10.1016/S1474-4422(08)70259-X.

[9] Amato MP, Portaccio E, Goretti B, Zipoli V, Hakiki B, Giannini M, et al. Cognitive impairment in early stages of multiple sclerosis. Neurological Sciences 2010;31:211–4. https://doi.org/10.1007/s10072-010-0376-4.

[10] MS Society. MS symptoms and signs 2019. https://www.mssociety.org.uk/about-ms/signs-and-symptoms (accessed August 29, 2019).

[11] MS Society. About fatigue 2019. https://www.mssociety.org.uk/about-ms/signs-and-symptoms/fatigue/about-fatigue (accessed August 29, 2019).

[12] Fraser RT, Johnson EK, Clemmons DC, Getter A, Johnson KL, Gibbons L. Vocational rehabilitation in multiple sclerosis (MS): A profile of clients seeking services. Work 2003;21:69–76.

[13] De Dios Pérez B, das Nair R, Radford K. Development of a Job Retention Vocational Rehabilitation Intervention for People with Multiple Sclerosis Following the Person-Based Approach. Clin Rehabil 2024. https://doi.org/10.1177/02692155241235956.

[14] De Dios Pérez B, Das Nair R, Radford K. A mixed-methods feasibility case series of a job retention vocational rehabilitation intervention for people with multiple sclerosis. Disabil Rehabil 2023:1–12. https://doi.org/10.1080/09638288.2023.2181411.

[15] De Dios Perez B, Holmes J, Elder T, Lindley R, Evangelou N, das Nair R, et al. Implementing vocational rehabilitation for people with multiple sclerosis in the UK National Health Service: a mixed-methods feasibility study. Disabil Rehabil 2024:1–13. https://doi.org/10.1080/09638288.2024.2417031.

[16] De Dios Perez B, Booth V, das Nair R, Evangelou N, Hassard J, Ford HL, et al. A qualitative study exploring how vocational rehabilitation for people with multiple sclerosis can be integrated within existing healthcare services in the United Kingdom. BMC Health Serv Res 2024;24:995. https://doi.org/10.1186/s12913-024-11424-y.

[17] Skivington K, Matthews L, Simpson SA, Craig P, Baird J, Blazeby JM, et al. A new framework for developing and evaluating complex interventions: Update of Medical Research Council guidance. The BMJ 2021;374:n2061. https://doi.org/10.1136/bmj.n2061.

[18] Eldridge SM, Chan CL, Campbell MJ, Bond CM, Hopewell S, Thabane L, et al. CONSORT 2010 statement: extension to randomised pilot and feasibility trials. BMJ 2016;355. https://doi.org/10.1136/BMJ.I5239.

[19] Ware JE, Sherbourne CD. The MOS 36-item Short-Form Health Survey (SF-36) I. Conceptual Framework and Item Selection 1992;6:473–83.

[20] Reilly MC, Zbrozek AS, Dukes EM. The Validity and Reproducibility of a Work Productivity and Activity Impairment Instrument. Pharmacoeconomics 1993;4:353–65. https://doi.org/10.2165/00019053-199304050-00006.

[21] Aarts J, Saddal SRD, Bosmans JE, de Groot V, de Jong BA, Klein M, et al. Don’t be late! Postponing cognitive decline and preventing early unemployment in people with multiple sclerosis: a study protocol. BMC Neurol 2024;24:1–15. https://doi.org/10.1186/S12883-023-03513-Y/FIGURES/1.

[22] Van Der Mei I, Thomas S, Shapland S, Laslett LL, Taylor B V., Huglo A, et al. Protocol for a pragmatic randomised controlled feasibility study of MS WorkSmart: an online intervention for Australians with MS who are employed. BMJ Open 2024;14:e079644. https://doi.org/10.1136/BMJOPEN-2023-079644.

[23] The EuroQol Group. EuroQol - a new facility for the measurement of health-related quality of life. Health Policy (New York) 1990;16:199–208. https://doi.org/10.1016/0168-8510(90)90421-9.

[24] Takasaki H, Chien CW, Johnston V, Treleaven J, Jull G. Validity and reliability of the perceived deficit questionnaire to assess cognitive symptoms in people with chronic whiplash-associated disorders. Arch Phys Med Rehabil 2012;93:1774–81. https://doi.org/10.1016/j.apmr.2012.05.013.

[25] Mapi Research Trust. Perceived Deficit Questionnaire: Scaling and Scoring. 2017. https://doi.org/10.4135/9781412984409.n14.

[26] Sullivan MJL, Edgley K, Dehoux E. A Survey of Multiple Sclerosis: Perceived Cognitive Problems and Compensatory Strategy Use. Canadian Journal of Rehabilitation 1990;4:99–105.

[27] D’Souza E. Modified Fatigue Impact Scale - 5-item version (MFIS-5). Occup Med (Chic Ill) 2016;66:256–7. https://doi.org/10.1093/occmed/kqv106.

[28] Chen G, Gully SM, Eden D. Validation of a New General Self-Efficacy Scale. Organ Res Methods 2001;4:62–83. https://doi.org/10.1177/109442810141004.

[29] Spitzer RL, Kroenke K, Williams JBW, Löwe B. A brief measure for assessing generalized anxiety disorder: The GAD-7. Arch Intern Med 2006;166:1092–7. https://doi.org/10.1001/archinte.166.10.1092.

[30] Kroenke K, Strine TW, Spitzer RL, Williams JBW, Berry JT, Mokdad AH. The PHQ-8 as a measure of current depression in the general population. J Affect Disord 2009;114:163–73. https://doi.org/10.1016/J.JAD.2008.06.026.

[31] Turner-Stokes L. Goal attainment scaling (GAS) in rehabilitation: a practical guide. Clin Rehabil 2009;23:362–70. https://doi.org/10.1177/0269215508101742.

[32] Matolić T, Jurakić D, Greblo Jurakić Z, Maršić T, Pedišić Ž, Paul Morrey C, et al. Development and validation of the EDUcational Course Assessment TOOLkit (EDUCATOOL)-a 12-item questionnaire for evaluation of training and learning programmes OPEN ACCESS EDITED BY 2023;8:1314584. https://doi.org/10.3389/feduc.2023.1314584.

[33] Avery KNL, Williamson PR, Gamble C, Francischetto EOC, Metcalfe C, Davidson P, et al. Informing efficient randomised controlled trials: exploration of challenges in developing progression criteria for internal pilot studies. BMJ Open 2017;7:e013537. https://doi.org/10.1136/BMJOPEN-2016-013537.

[34] Hooper R. Justifying sample size for a feasibility study. London: n.d.

[35] Julious SA. Sample size of 12 per group rule of thumb for a pilot study. Pharm Stat 2005;4:287–91. https://doi.org/10.1002/pst.185.

[36] Lewis M, Bromley K, Sutton CJ, McCray G, Myers HL, Lancaster GA. Determining sample size for progression criteria for pragmatic pilot RCTs: the hypothesis test strikes back! Pilot Feasibility Stud 2021;7:1–14. https://doi.org/10.1186/S40814-021-00770-X/FIGURES/2.

[37] Braun V, Clarke V. Using thematic analysis in psychology. Qual Res Psychol 2006;3:77–101. https://doi.org/10.1191/1478088706qp063oa.

[38] Fereday J, Muir-Cochrane E. Demonstrating Rigor Using Thematic Analysis: A Hybrid Approach of Inductive and Deductive Coding and Theme Development. Int J Qual Methods 2017;5:80–92. https://doi.org/10.1177/160940690600500107.

[39] Byrne D. A worked example of Braun and Clarke’s approach to reflexive thematic analysis. Qual Quant 2022;56:1391–412. https://doi.org/10.1007/S11135-021-01182-Y/FIGURES/D.
